# Treatment with low-dose tyrosine kinase inhibitors due to significant haematologic toxicity in patients with CML with prolonged treatment failure prevents haematologic progression

**DOI:** 10.1016/j.htct.2024.03.010

**Published:** 2024-07-22

**Authors:** Lucia Vráblová, Hana Klamová, Ivana Skoumalová, Jana Navrátilová, Romana Janská, Jan Grohmann, Milena Holzerová, Edgar Faber

**Affiliations:** a University Hospital Olomouc, Faculty of Medicine and Dentistry, Palacky University in Olomouc, Czech Republic; b Institute of Hematology and Blood Transfusion, Prague, Czech Republic

**Keywords:** Chronic myeloid leukaemia, Tyrosine kinase inhibitor, Low dosage, Intermittent dosage, Haematologic toxicity, Kinase domain mutation

## Abstract

**Background:**

A lower dosage of tyrosine kinase inhibitors (TKIs) in patients with chronic myeloid leukaemia (CML) has shown efficacy in managing short-term toxicity and maintaining a deep molecular response in patients who fail to achieve treatment-free remission.

**Method:**

From over 700 patients with CML who were treated at two centres over the last three decades, this retrospective study identified eight patients characterised by long-term treatment failure and simultaneous prolonged significant haematologic toxicity that prevented the use of the standard tyrosine kinase inhibitor dosage.

**Results:**

Patients had a high or intermediate ELTS risk score, and most had significant comorbidities. Two patients were treated previously with busulfan, and four were aged over 70, which might explain the reduced pool of normal haematopoietic stem cells. However, concomitant myelodysplastic syndrome or the presence of clonal haematopoiesis of indeterminate potential was not demonstrated. Despite prolonged treatment failure, the survival of these patients (who were ineligible for stem cell transplantation) ranged from 45-396 months. Neither mutations in the ABL kinase domain nor additional cytogenetic abnormalities developed during the treatment of these patients, prompting speculation about the low selective pressure of low-dose tyrosine kinase inhibitors and/or the absence of mutations at diagnosis.

**Conclusion:**

It is important not to stop treatment with tyrosine kinase inhibitors at a low personalised dosage in CML patients with prolonged significant haematologic toxicity despite long-term treatment failure.

## Introduction

Tyrosine kinase inhibitors (TKIs) have considerably improved the prognosis of patients with chronic myeloid leukaemia (CML). This has been achieved by their targeted mode of action that enables the competitive inhibition of leukaemic BCR::ABL1 tyrosine kinase and leads to the deepening of the response to treatment at the molecular level in the majority of patients. Consequently, the guidelines for CML treatment reflect this success by shifting the therapeutic goal to a major MR 3.0 and, more recently, to a deep MR 4.0-5.0 molecular response for at least some patients, with the aim of achieving treatment-free remission (TFR).^1^

The dosage of TKIs plays an important role both in the efficacy and toxicity of targeted CML treatment and has been the focus of many studies, some of which have led to changes in the recommended dosage of almost all TKIs.^2^, ^3^, ^4^, ^5^ Short-term changes in actual TKI dosage (with interruptions and reductions) are frequently used in the management of short-term TKI toxicity.^6^ Studies have shown that low-dose and intermittent dosage strategies may represent a relevant option not only for the management of haematologic toxicity but also for maintaining a deep molecular response, especially in the case of an unsuccessful attempt to achieve TFR.^7^, ^8^, ^9^, ^10^, ^11^, ^12^, ^13^ Herein, we show that in rare and frail patients ineligible for allogeneic haematopoietic stem cell transplantation (AHSCT) and compromised with prolonged haematologic toxicity and/or significant comorbidity, the low-dose or intermittent dosage of TKIs may be a useful approach to prevent progression to the blast phase despite prolonged treatment failure, according to the current guidelines.

## Methods

Patients 1 to 6 were treated at the Department of Hemato-Oncology at University Hospital Olomouc, and the remaining patients (Patients 7 and 8) were treated at the Institute of Haematology and Blood Transfusion (UHKT) in Prague. Response to treatment during the follow-up of the patients was assessed according to the valid version of the European LeukemiaNet (ELN) guidelines.^1^^,^^14^, ^15^, ^16^ The methods for cytogenetic and molecular monitoring have been described previously.^17^^,^^18^

Mutations in the ABL kinase domain were repeatedly examined by Sanger sequencing in all patients during the follow-up. After the introduction of next-generation sequencing (NGS) in UHKT Prague (2011), the mutational status of Patients 7 and 8 was routinely examined by the technique. Two samples (one from the latest follow-up) from Patients 1 to 6 were examined retrospectively by NGS in Prague.

Clonal haematopoiesis of indeterminate potential (CHIP) was assessed by NGS using HaloPlex target enrichment library preparation technology (Agilent). A small NGS ClearSeq AML panel covering 20 genes (*DNMT3A* [exons 4/8/13/15/16/18–20/22/23], *JAK2* [12/14], *MPL* [10], *ASXL1* [12], *EZH2* [8/17/18], *SF3B1* [13–15/17], *SRSF2* [1], *TET2* [3/9–11], *U2AF1* [2/6], *CSF3R* [14/17], *SETBP1* [3], *CBL* [8/9], *IDH1*/*2* [4], *RUNX1* [3/4/8], *NRAS* [2/3], *TP53* [5–8], *CEBPα* [1], *FLT3* [14/20] and *NPM1* 11]) that are frequently mutated in myeloproliferative disorders was applied. Pair-end sequencing was performed on a MiSeq (Illumina) system. The mutational profiles of Patients 2 to 6 were evaluated in SureCall, and the detected variants were annotated in the VarSome and Cosmic databases.

The toxicity of the treatment was graded according to version 5.0 of the Common Terminology Criteria for Adverse Events system of the US National Cancer Institute.^19^

The study was conducted in accordance with the Declaration of Helsinki and approved by the Institutional Review Boards. Informed consent was obtained from all subjects involved in the study.

## Results

**Figure 1:**
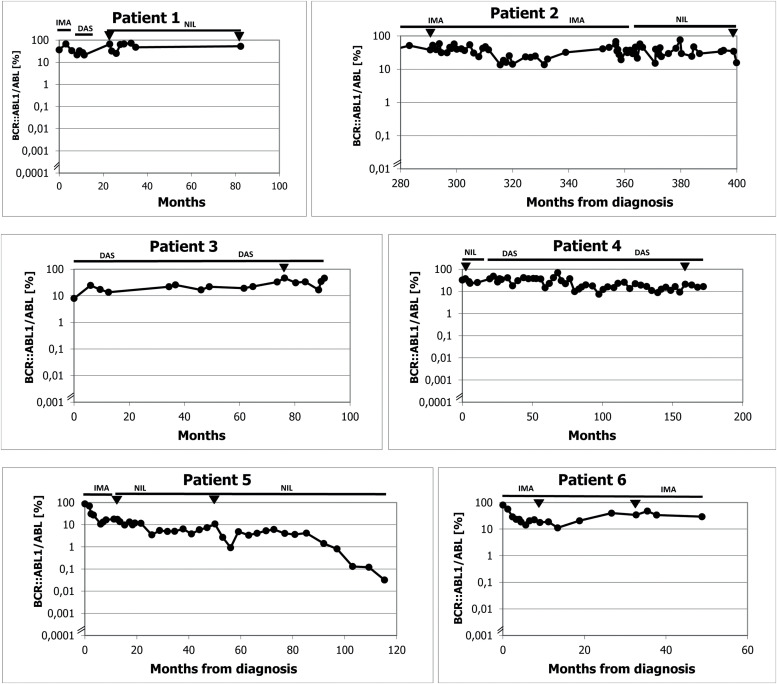

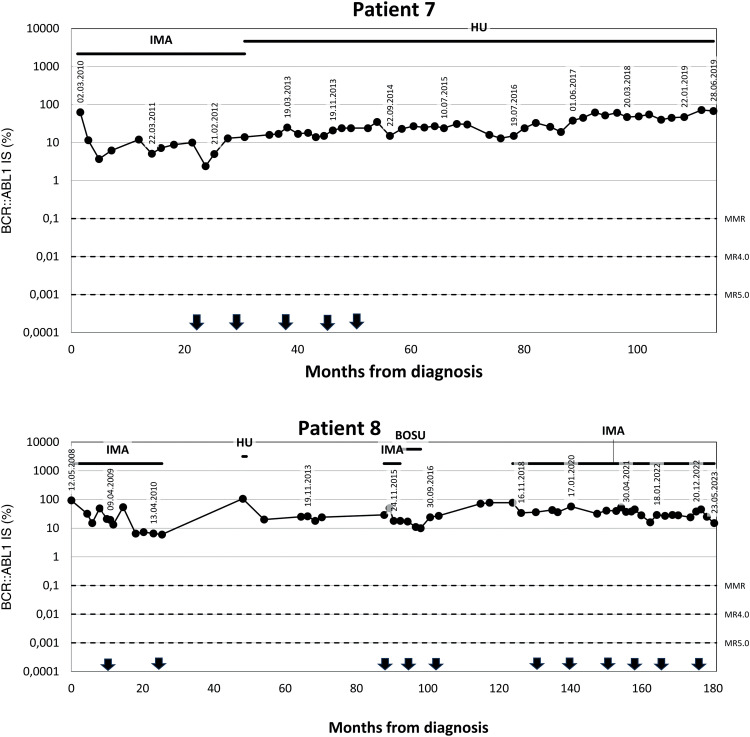
A/B Results of real time polymerase chain reaction (PCR) for BCR::ABL1 in individual patients. Figure 1A: Patients 1-6 from Olomouc. Triangles show the samples tested for ABL kinase mutations with negative results by the next generation sequencing in Prague by courtesy of Kateřina Machová Poláková. ABL kinase mutations were examined with negative results also using Sanger sequencing on many other occasions (samples are not indicated). In Patient 1 the time on axis A shows the interval of the treatment with TKI only. The follow-up of Patient 2 during the first 280 months was performed partly at another center and partly the examinations were by cytogenetics and fluorescence *in situ* hybridization (FISH) only. Earlier examinations in Patients 3 and 4 were also performed by cytogenetics and FISH. Figure 1B: Patients 7 and 8 from Prague. Arrows show the samples tested for ABL kinase mutations with negative results by the next generation sequencing. Abbreviations: BOSU: bosutinib; DAS: dasatinib; HU: hydroxyurea; IMA: imatinib; NIL: nilotinib.

**Figure 2:**
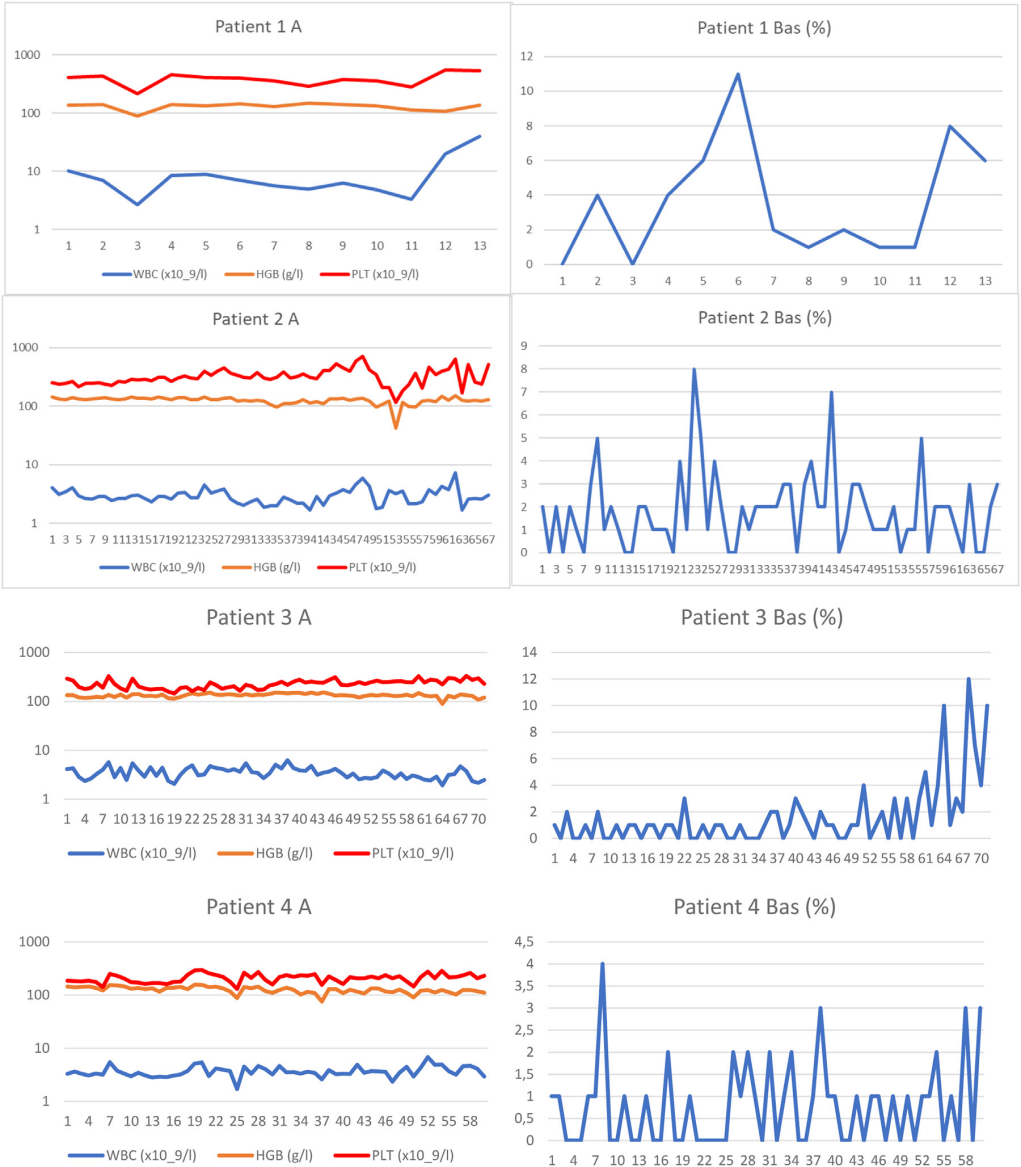

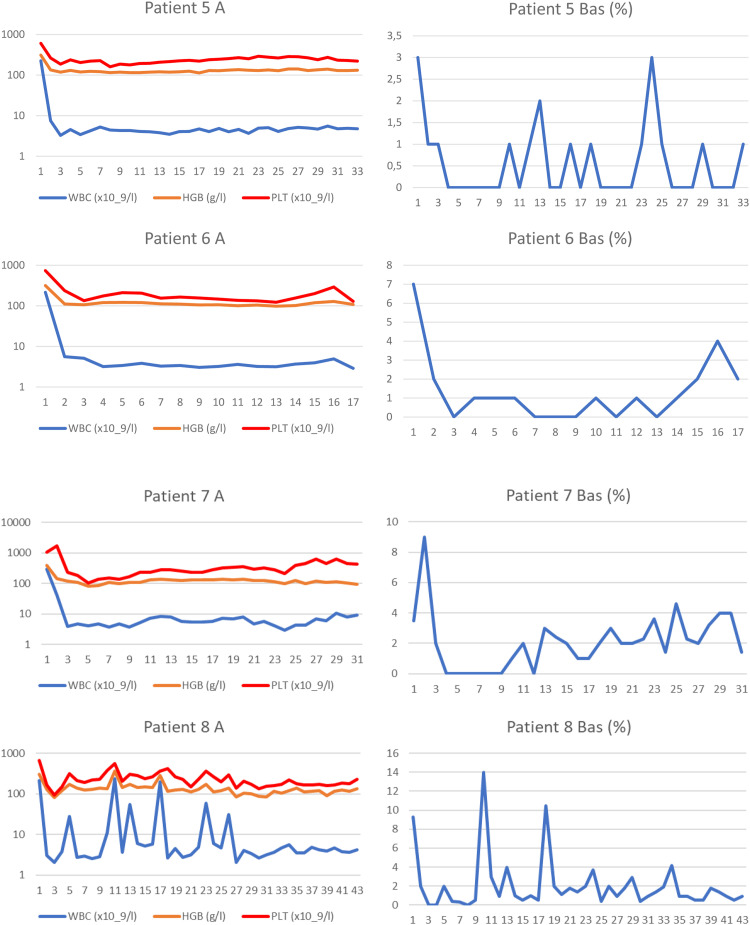
A: White blood cell (WBC) and platelet counts and haemoglobin levels of each patient are reported from samples taken dominantly at the time of real-time polymerase chain reaction (PCR) testing. Logarithmic scale is used on axis y in order to clearly show the WBC counts. B: Basophil percentage trends are shown. Numbers of the samples (not the time) are given on axis x. Trends in basophils in Patients 1, 2, 3 and 8 together with WBC count trend of Patient 8 show periods of haematological progression after prolong interruptions in TKI treatment. Periods with most severe haematological toxicity may not be recorded in cases that they did not occur at the time of real-time PCR testing.

We identified eight patients from among over 700 patients with CML who had been treated with TKIs at both cooperating centres since the introduction of imatinib for clinical use. These patients were characterized by prolonged significant haematologic toxicity (at least grade 3 thrombocytopenia and/or grade 3 neutropenia) after the standard TKI dosage made its continuation or reintroduction impossible. After experiencing haematologic toxicity, the TKI dosage was individually reduced and even interrupted on many occasions. The clinical and laboratory characteristics of the patients are outlined in Table 1, and the results of real-time polymerase chain reaction (PCR) are given in Figure 1. The results of blood counts (white blood cell count [WBC], haemoglobin, platelet count and percentage of basophils from the WBC differential count) are depicted in Figure 2. Generally, the patients were elderly, with significant comorbidities that, in most cases, made AHSCT impossible. These patients had an increased risk of CML, and three of them were treated with interferon before the introduction of imatinib. Two patients had a history of busulfan therapy. The interval from diagnosis to initiation of TKI treatment varied in the patients considerably. Half of the patients had a long interval while the remaining patients started the treatment immediately after diagnosis. Changes in their treatment were considered according to the present availability of TKIs and the valid guidelines. Tests for ABL kinase mutations produced repeatedly negative results in all patients. Cytogenetic examinations of marrow aspirate were performed on most of the patients (some refused), at least at the first episode of treatment resistance. The plasma levels of TKIs were examined in all patients. Trough levels were not measured in each individual due to the routine clinical setting, with the time of TKI administration adjusted to each patient's preference. Despite this limitation, the results were not consistent with the abnormal pharmacodynamics of TKIs (data not shown). Patients 2, 3 and 4 were also included in our first report on intermittent imatinib treatment as Patients 4, 5 and 12, respectively.^7^

**Table 1:** Clinical, laboratory, treatment and survival data of the patients.

Patient number, gender (f, m)	Age at diagnosis	Sokal / ELTS risk score	BCR::ABL transcript	Treatment before TKIs	Interval from diagnosis to initiation of TKI treatment (months)	Sequence of TKIs with the dosage used most of the time	Molecular response best / worse (Q-PCR IS %)	Haematologic toxicity	Duration of the TKIs treatment / duration of low-dose TKI treatment (months)	Comorbidity	Survival from diagnosis (months), cause of the death
1 m	37	Unknown	b2a2	HU	Approx. 48	IMA (400mg/D) – DAS (100mg/D; 100mg/5DW; 100mg/4DW) – NIL (400mg D, 2-5DW; 300mg D, 2DW) + HU	21.2 / 72.72	ANC gr 4; PLT gr 3; anaemia gr 4	82 / 71	Morbus Bechterev, Somatic asthenia, Miliary tuberculosis	108 blast phase of CML
2 f	17	IM / IM	b3a2	BU, INF, HU	Approx. 135	IMA (600mg/D; 400mg/1DW, 100mg/D) – NIL (800mg/D; 400mg/D)	13.38 / 57.57	ANC gr 4; PLT gr 3; anaemia gr 4	264 / 261	Addiction to psychopharmacs, Epilepsy	396 alive
3 f	56	HI / HI	b2a2	HU, mini-ICE, Hi-D BU+ASCT, INF	33	IMA (400mg/D; 400mg 1-4DW) – DAS (40mg/D; 50mg 2-5DW) – NIL (400mg/D) – DAS (70mg 3DW)	8.0 / 46.56	ANC gr 4; PLT gr 4; anaemia gr 2	245 / 243	Chronic bronchitis, Peripheral artery ischemic disease, Carotid artery stenosis Acute biliary pancreatitis	279 alive
4 f	55	HI / IM	b3a2	HU, INF	15	IMA (400mg/D, 400mg 1-5DW) – DAS 50-70mg 3-5DW) – NIL (400mg 3-5DW) – DAS (60mg 3DW)	7.47 / 70.23	ANC gr 3; PLT gr 3; anaemia gr 2	218 / 216	CAD MI, Hypertension, Transitory cerebral ischemia	233 alive
5 m	80	HI / HI	b3a2	HU	0.5	IMA (200-300mg/D 400mg/D) NIL (400mg/D; 400mg 2-3DW)	0.12/ 30.74	ANC gr 3; PLT gr 3; anaemia gr 3	110 / 106	Peripheral artery ischemic disease, Pulmonary emphysema	110 alive
6 f	80	HI / HI	b3a2	HU	0.3	IMA (200-400mg/D, 400mg 2DW)	11.17 / 56.49	ANC gr 2; PLT gr 4; anaemia gr 2	45 / 44	Hypertension Dissecting aortic aneurysm, Alzheimer dementia	45 alive
7 f	70	HI / IM	b3a2	HU	1	IMA (400mg/D, 200mg/D, 100mg/D, 300mg/D, 200mg/D) – HU	2.4 / 72.0	ANC gr 4; PLT gr 3; anemia gr 2	30 / 21	Hypertension, Diabetes mellitus	118 Heart failure, Incipient acceleration of CML
8 m	30	IM / IM	b3a2	HU	0.6	IMA (400mg/D, 300mg/D, 400mg/D, 200mg/D)	6.0 / 77.0	ANC gr 3; PLT gr 4; anaemia gr 2	175 / 140	Renal failure, Nephrotic syndrome, Combined thrombophilia (APCR, protein S deficiency), Hypacusia	176 alive

Legend: TKI: tyrosine kinase inhibitor; m: male; f: female; IM: intermediate; HI: high; HU: hydroxyurea; BU: busulfan; INF: interferon alpha; HI-D BU+ASCT: high-dose busulfan with autologous peripheral stem cell transplantation; dosage of TKI: xx mg; D: xx mg daily; 4DW: 4 days weekly; 1-3DW: 1-3 days weekly; ANC: absolute neutrophil count; PLT: platelet count; gr: grade; CAD: coronary artery disease; MI: myocardial infarction; APCR: activated protein C resistance.

### Comments on the individual patients

The initial characteristics of CML in Patient 1 are unknown. He was a foreigner who immigrated to the Czech Republic and was referred to our center in 2006 in the chronic phase of the disease with a probable four-year duration and was treated with hydroxyurea. He was an asthenic heavy smoker and because of morbus Bechterew (ankylosing spondylitis), he was addicted to analgesics. Initially, he refused marrow aspiration. His treatment was switched to the standard dose of imatinib, which did not lead to any cytogenetic response (measured by fluorescence *in situ* hybridization – FISH – of peripheral blood), and after ten months of treatment, the therapy was changed to dasatinib, initially given at a daily dose of 100 mg. The patient developed pleural effusion, and thereafter the dose was reduced to 100 mg four days per week. The treatment resulted in severe pancytopenia (WBC: 0.75 x 10^9^/L; absolute neutrophil count [ANC]: 0.45 x 10^9^/L; haemoglobin [Hb]: 66 g/L; platelets [PLT]: 28 x 10^9^/L) complicated by miliary tuberculosis four months after dasatinib initiation. After recovery achieved by growth factors, platelet transfusions and tuberculostatic treatment with a reduced dose of nilotinib (200 mg twice daily) were initiated ten months after stopping dasatinib. Treatment with dasatinib was not resumed because of a possible immune suppressive effect, despite the drug leading to probably the best therapeutic response (see Figure 1 for the molecular response to the treatment). After four years, the patient moved to another centre, where TKI treatment was stopped in October 2013 (82 months after starting imatinib) because of treatment failure and alleged poor compliance with treatment. He temporarily returned to our centre, and low-dose nilotinib treatment was resumed for 16 months without a hematologic response. The patient was managed with hydroxyurea at a local centre and died after the blast phase nine years after our initial examination. An NGS analysis was not performed on this patient because of the unavailability of DNA.

Patient 2 (Patient 4 in our previous publication^7^) was diagnosed in 1989 when interferon was not available and bone marrow transplantation was not indicated because of the lack of a family donor. Despite her age, the patient was treated with busulfan, which, in our opinion, was later responsible for her poor haematologic tolerance of TKIs. At the time when unrelated transplantation programs were initiated in transplant centres in Czechia, there was disagreement about the indication because of the fear of a higher risk of transplant-related mortality due to the long interval since diagnosis. Later, the patient refused transplantation. At the time of imatinib availability, she started treatment with a full dose of 400 mg daily, but the therapy had to be interrupted after two months because of hematologic toxicity (WBC: 0.9 x 10^9^/L; ANC: 0.1 x 10^9^/L; Hb: 96 g/L; PLT: 8 x 10^9^/L). After recovery of the blood count, the treatment was resumed at a dose of 100 mg daily, but we felt that she might benefit from a standard dose given only for a few days per week with the aim of achieving therapeutic plasma levels, at least temporarily, so she was given 400 mg once to twice a week. Consecutive TKI changes did not improve her response, which ranged between 13.38% and 76.07% in real-time PCR (see Figure 1). An NGS analysis performed on a marrow sample taken in April 2019 revealed a frameshift deletion in the *ASXL1* gene (ASXL1 [p.E635Rfs*15], c.1900_1922del, COSV60102280, NM_015338.6; VAF = 16%). The presence of this mutation was not confirmed in the CD3^+^ lymphocyte fraction, and CHIP was excluded.

Patient 3 (Patient 5 in our previous publication^7^) was recruited to a prospective randomized European Society for Blood and Marrow Transplantation (EBMT) 99 study with autologous peripheral stem cell transplantation. She was randomized to the transplant arm. Peripheral stem cells were collected after priming with mini-ICE (ifosfamide, carboplatin, and etoposide phosphate) and transplanted after conditioning with high-dose busulfan in May 2000. After engraftment, the patient was treated briefly with interferon-alpha, but this had to be stopped because of depression. Significant haematologic toxicity had been observed previously after interferon. Imatinib was started immediately after registration of the drug and approval for health insurance reimbursement in the Czech Republic, 33 months after diagnosis. The treatment was started with a full dose; however, a reduction of the dose was necessary because of haematologic toxicity and problems with the patient's gastrointestinal toleration of imatinib. After four years of therapy with imatinib, she was switched to dasatinib because of failure to achieve a significant cytogenetic response (60% BCR::ABL1 in FISH). Again, the dose of dasatinib had to be reduced due to haematologic toxicity. After 41 months of dasatinib treatment, nilotinib was given because of cytogenetic progression; however, the treatment had to be stopped because of peripheral arterial ischaemia, and the patient was switched back to dasatinib. After an interruption in therapy for one month in connection with surgery for carotid artery stenosis in February 2022, the patient progressed to the chronic phase, with 18% basophils in her peripheral blood. Haematologic remission was regained by dasatinib given at a reduced dosage together with repeated applications of granulocyte colony stimulating factor (G-CSF). However, the response has not been maintained, and recently she was switched to bosutinib. Response to bosutinib has not been evaluated yet because of the short duration of the treatment.

Patient 4 (Patient 12 in our previous publication^7^) was treated with the standard dose of imatinib after one year of interferon therapy because of cytogenetic failure. The dose of imatinib had to be reduced as well as that of dasatinib, which was given after 22 months because of the cytogenetic failure of imatinib. The best cytogenetic response after dasatinib was 25% in FISH. Nilotinib was given at a dose of 400 mg daily with the aim of improving the cytogenetic response; however, because of cytogenetic deterioration, the patient was switched back to dasatinib. At a lower dosage and with the occasional interruption and treatment with G-CSF, the patient has remained on this medication, maintaining an approximately major cytogenetic response. An NGS analysis performed in May 2020 revealed a missense variant of unknown significance in the *EZH2* gene (EZH2 [p.V679L], c.2035G/C, NM_004456.5, VUS; VAF = 14%) localized in the highly conserved SET domain of the EZH2 protein. This variant is yet to be described and annotated.

Patient 5 started low-dose imatinib therapy, which was gradually increased to a full daily dose; however, the best molecular response was 17%, and after 14 months the treatment was switched to nilotinib at 400 mg daily. The dosage had to be reduced because of significant haematologic toxicity and the worsening of peripheral artery ischaemia. After eight years of treatment with a reduced dose (400 mg daily for two days each week), the molecular results approached the level of a major molecular response, while the ischaemia of the lower extremities was stabilized.

Patient 6 was treated for the entire duration with a lower imatinib dosage from diagnosis because of her advanced age, comorbidity and finally, because of significant thrombocytopenia. The thrombocytopenia also forced several interruptions in treatment. The patient refused bone marrow aspiration, which was indicated in an attempt to confirm myelodysplastic syndrome (MDS). Presently, despite the deterioration with Alzheimer's dementia, she maintains a molecular response to the treatment at a level of 33.89-47.42% at a dose of 400 mg of imatinib two days per week.

Patient 7 commenced treatment with a standard dose of imatinib, but this had to be reduced and interrupted repeatedly. Finally, after 30 months of imatinib treatment, the patient was switched to hydroxyurea at the age of 73 on her own decision. The treatment with hydroxyurea lasted a further seven years before she died of cardiac failure with simultaneous acceleration of CML.

Patient 8 started treatment with the standard dose of imatinib, but this had to be reduced to 300 mg daily due to Grade 3 haematologic toxicity after three months of therapy. After two years of reduced treatment, the patient was temporarily lost to follow-up and returned three years in the accelerated phase of CML. With imatinib given at 400 mg/day, the chronic phase was restored, but after two months of treatment, severe haematologic toxicity occurred, so the dose was reduced to 200 mg daily. Even at this dose, the treatment had to be interrupted. Bosutinib was given shortly in an attempt to overcome the resistance, however it had to be stopped early because of intolerance. Communication with the patient was hampered by his severe hypacusia; also, poor compliance was highly suspected.

## Discussion

The recommended dosage of TKIs has been subject to revision in Phase 3 studies. This has led to the adjustment of the recommended dose of nilotinib, dasatinib, bosutinib and ponatinib.^2^, ^3^, ^4^, ^5^ Primarily, the dose of TKIs has been reduced, which is associated with improved tolerance of the drug while preserving efficacy. On the other hand, the recommended dose of imatinib has remained at 400 mg daily despite evidence from national academic studies showing some improvement in the response of patients treated with 800 mg per day.^20^ A lower-than-recommended TKI dose should not be routinely used because of the risk of developing resistance.^1^ Conversely, pharmacodynamic studies have shown that in the case of imatinib, a drug level sufficient for the *in vitro* inhibition of leukaemic cell growth may be achieved with a dose of 200 mg daily.^21^

In our previous report, we showed that the intermittent dosage of imatinib may safely be used for the management of short-term haematologic toxicity.^7^ Most patients from this first study overcame the period of toxicity and were subsequently treated with full-dose imatinib or other TKIs, but some needed continual reduction of the TKI dosage or the use of an intermittent schedule due to persistent significant haematologic toxicity. Three patients (Patients 4, 5 and 12) from the first report were included in the present study (Patients 2, 3 and 4, respectively).

A lower TKI dosage is an accepted strategy when haematologic toxicity lasts for a few weeks; however, according to the guidelines, short-term interruption of the treatment together with the application of growth factors in neutropenia or transfusion replacement in anaemia or thrombocytopenia is the preferred management strategy.^1^^,^^6^^,^^14^, ^15^, ^16^ Significant haematologic toxicity related to TKIs is typically observed during the first months of treatment and usually does not persist for many months or years, as in this report. Simultaneous MDS may cause such long-term cytopenia. Significant dysplastic morphological features or typical MDS cytogenetic findings in Ph-negative mitoses were not observed in follow-up marrow aspirations from these patients. The long interval between diagnosis and initiation of TKI treatment was one probable factor influencing the response in half of the patients, but the second half of the patients started TKI treatment early after diagnosis. Reduced marrow capacity may be linked to busulfan in Patients 2 and 3. On the other hand, Patients 5, 6 and 7 were older, wherein the regeneration capacity of the bone marrow may be compromised by CHIP. Mutations in DTA genes (*DNMT3A, TET2* and *ASXL1*) cause approximately 70% of all mutations in CHIP.^22^ NGS testing revealed one patient (Patient 2) with the deletion of 23 bp in the *ASXL1* gene, causing a frameshift, and one patient (Patient 4) with a variant of unknown significance in the *EZH2* gene. These mutations may have participated in treatment failure.

However, detailed testing (confirmation of mutations in the CD3^+^ population) excluded CHIP in patients with proven mutations. There were no mutations in the remaining three patients from Olomouc (Patients 3, 5 and 6). Mutations in ABL kinase domain were not identified in any of the patients during the course of the treatment. Sanger sequencing was used for mutation detection in Olomouc but it was supplemented by NGS on two occasions in all patients, leading to the confirmation of negative results. However, bearing in mind the high level of residual disease in all patients, Sanger sequencing should be sufficiently sensitive in order to detect mutated clones causing treatment resistance.

Some studies have shown that low-dose or intermittent-dose TKIs may play a role in the maintenance of major or deep molecular responses in patients who progress after an unsuccessful attempt to stop TKI therapy to achieve TFR.^8^^,^^9^^,^^12^^,^^23^^,^^24^ Therefore, low-dose TKIs are attracting increasing attention, as shown in recent reviews.^25^^,^^26^ In addition, the efficacy of low-dose TKIs was confirmed by computer modelling.^27^ However, low-dose TKI treatment has not been reported to be used in the case of treatment failure. In such a situation, a change to next-generation TKIs based on the results of ABL kinase mutation testing and the exclusion of additional cytogenetic abnormalities in Ph-positive cells is recommended. After the failure of two or more TKIs, the feasibility of AHSCT should be determined.^1^ However, in the patients of this study, ABL kinase mutations that may lead to the targeted selection of TKIs were not present and AHSCT was not feasible because of their advanced ages, comorbidities or refusal. We always tried to start a TKI dose at the standard level and in the case of switches in treatment. The exceptions were Patients 5 and 6, in whom, because of their advanced ages and comorbidities, treatment was started at a lower than the standard recommended dose with the aim of increasing the dose according to their tolerance to therapy. G-CSF was used in the situation of severe neutropenia, but we were concerned with the prolonged use of growth factors during simultaneous treatment failure. The TKI dose and treatment interruptions were adjusted individually either to achieve a standard dose with interruptions or a lower dose without them. The progression of CML in three patients (Patients 1, 3 and 8) after prolonged (longer than one month) interruptions in TKI treatment or a switch to hydroxyurea highlights the need for both close patient monitoring and TKI treatment continuation. Furthermore, it shows that under the condition of treatment failure, interruptions in treatment should be as short as possible, usually at the trough of significant haematologic toxicity. With such an approach, even an optimal response can be achieved in some patients, e.g. Patient 5, who approached a major molecular response after low-dose nilotinib treatment (400 mg two days per week) only after nearly ten years of treatment. Such a response was not achieved by the other patients in this cohort.

The group of patients in the present study was too small to compare individual TKIs in a clinical setting of prolonged treatment failure. Dasatinib exerted a better response in those patients who progressed with nilotinib treatment (Patients 4 and 7). The reason for this can only be hypothesized: a wider range of inhibition targets, including Src tyrosine kinase and others that play a role in the immune response. On the other hand, this effect may be responsible for the severe infection (miliary tuberculosis) in Patient 1. Treatment with a more potent TKI (e.g. ponatinib) was frequently not indicated because of comorbidities. We believe that the reduced haematological reserves were more important in treatment resistance by precluding full dose therapy in these patients than the failure of individual TKI to sufficiently suppress leukaemic cells.

In CML, clonal progression with the formation of new mutations in the ABL kinase domain is assumed to develop under the selective pressure of TKI treatment, and patients with chronic-phase CML are very unlikely to harbour resistant mutations at diagnosis.^28^ Conversely, it was demonstrated that high-risk CML possess additional somatic genetic changes in addition to BCR::ABL1 at diagnosis.^29^ It may be speculated that a very limited number of mutated clones or single mutated stem cells present at the diagnosis of CML may simply escape laboratory testing. Normal-dose TKI treatment may thereafter select this clone, causing progression within the first months after diagnosis, or in the case of isolated affected stem cells, it may cause a relapse after several years of dormancy. Our clinical observations that rare CML patients with long-term (up to 20 years) failure of TKI treatment and a significant residual leukemic population (30-70% of BCR::ABL1 positivity in real-time PCR) did not experience hematologic progression or develop additional cytogenetic changes or ABL kinase mutations over long periods is surprising. Does this mean that mutations in the ABL kinase domain should be present only at diagnosis and not develop during treatment? Does it mean that our patients had biologically benign leukaemia lacking clonal diversification at diagnosis? Does it mean that low or intermittent TKI dosage lacks sufficient selective pressure for mutational development? The answers to these questions are unknown.

Our group of patients probably represents less than 1% of routinely treated CML patients, so it cannot justify any generalizations, changes in treatment strategy or recommendations.

## Conflicts of interest

We confirm that there are no known conflicts of interest associated with this publication and there has been no significant financial support for this work that could have influenced its outcome. EF declared support not associated with the preparation of the manuscript: Novartis – supported lectures, member of the local advisory board; Angelini – supported lectures; Zentiva – supported lectures, member of the advisory board; ELN Foundation – travel support, HPST – travel support.
